# Alovudine Targets mtDNA-dependent Proteins Encoded by Genes Recurrently Amplified in Metastatic Breast Cancer Cohorts

**DOI:** 10.1016/j.mcpro.2026.101622

**Published:** 2026-07-14

**Authors:** Jessica Brandi, Gloria Gatto, Federica Sotgia, Michael P. Lisanti, Daniela Cecconi

**Affiliations:** 1 Department of Biotechnology, University of Verona, Verona, Italy; 2 Lunella Biotech, Ottawa, Ontario, Canada; 3 Translational Medicine, School of Science, Engineering and Environment (SEE), University of Salford, Greater Manchester, United Kingdom; 4 The Institute of Mental Health Research (IMHR), University of Ottawa, The Royal Ottawa Hospital (ROH), Ottawa, Ontario, Canada; 5 Département de Médecine, Université Laval, Québec City, Canada

**Keywords:** laovudine, breast cancer, metastasis, mitochondria, mtDNA-depleted cells, mtDNA, proteomics

## Abstract

Previously, we demonstrated that high levels of mitochondrial DNA (mtDNA) were functionally associated with “stemness” and aggressive phenotypic behaviors in human breast cancer cells, including spontaneous metastasis. More specifically, we showed that treatment with Alovudine induced mtDNA-depletion in MDA-MB-231 cells and prevented their ability to form colonies *in vitro* and metastasize *in vivo*. To better understand the underlying mechanism(s) and identify candidate mtDNA-dependent mitochondrial protein biomarkers relevant to metastatic breast cancer, Alovudine-treated MDA-MB-231 cells were subjected to proteomics analysis. For comparison purposes, mtDNA-depleted cells (MDA-MB-231 and MCF-7) were generated and also subjected to proteomics analysis. Intersection of these three distinct data sets revealed that a small number of proteins were commonly downregulated in mtDNA-depleted cells and Alovudine-treated cells. Remarkably, many of these nuclear-encoded mitochondrial genes (>20) exhibited recurrent genomic amplification across metastatic breast cancer cohorts. Therefore, the action of a single drug, namely Alovudine, was sufficient to effectively suppress the expression of a large number of mitochondrial proteins associated with i) gene amplification and ii) cancer cell metastasis, in human breast cancer patients. These findings may have important clinical implications for the development of new therapeutics targeting advanced breast cancer.

## Highlights

• Alovudine suppresses mitochondrial gene expression linked to metastasis.
• Proteomics reveals shared down‑regulated proteins in mtDNA-depleted and treated cells.
• Amplified mitochondrial genes in metastasis are inhibited by Alovudine.

## Graphical Abstract

**Figure:**
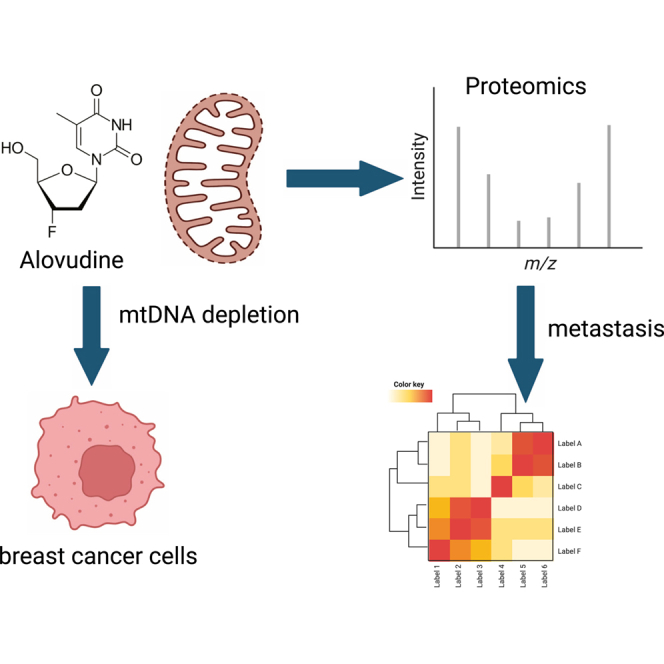

## In Brief

We used comparative proteomics to dissect the molecular consequences of mtDNA depletion in breast cancer cells, distinguishing drug-specific effects from mitochondrial loss. mtDNA depletion consistently suppressed OXPHOS proteins and rewired mitochondrial pathways, while Alovudine induced broader metabolic, cell cycle, and metastasis-associated changes. Notably, several mtDNA-dependent proteins downregulated in vitro are genomically amplified in metastatic tumors, linking mitochondrial function to proteome remodeling and metastatic potential.

The multifaceted interplay between mitochondrial function and cancer progression has emerged as a central theme in modern oncology (1). Beyond their canonical role in ATP production via oxidative phosphorylation (OXPHOS), mitochondria govern a wide array of cellular processes, including programmed cell death, redox regulation, calcium signaling, and metabolic reprogramming. Their dynamic nature, encompassing fission, fusion, and mitophagy, enables rapid adaptation to environmental and intracellular cues. Structurally complex and genetically distinct, mitochondria encode key components essential for cellular viability and stress responses.

In the context of tumor biology, these organelles act as integrative hubs that coordinate key pathways underpinning malignancy. Crucially, mitochondrial function has been implicated in maintaining cancer cell stemness, promoting metabolic flexibility, and mediating resistance to standard therapeutic interventions (2). Notably, our previous studies in breast cancer have highlighted the role of mitochondrial biogenesis in enhancing tumor growth and conferring resistance to autophagy (3), supporting anchorage-independent survival and expansion of cancer stem-like cells (CSCs) (4), and promoting stemness through mitochondrial fission (5). More recently, we demonstrated that inhibiting mitochondrial DNA (mtDNA) synthesis with Alovudine significantly reduced spontaneous metastasis formation while exerting only a modest effect on primary tumor growth (6). Despite these insights, the specific mitochondrial proteins involved in metastatic progression remain unidentified.

Here, using a proteomic approach, we demonstrate that Alovudine treatment of MDA-MB-231 breast cancer cells leads to a marked downregulation of mitochondrial proteins, mirroring the suppression observed in mtDNA-depleted cells (7, 8) and underscoring their dependence on mtDNA. Notably, many of these proteins are encoded by genes showing recurrent genomic amplification across independent breast cancer cohorts, including metastatic breast cancer cohorts.

These observations suggest that mtDNA-dependent proteins may represent promising candidate biomarkers and therapeutic targets in advanced breast cancer and further support the notion that targeting mtDNA could represent a novel therapeutic strategy.

## Experimental Procedures

### Experimental Design and Statistical Rationale

Proteomic profiling was performed on biological replicates (n = 4 per group) using LC-MS/MS with label-free quantification. Differentially expressed proteins (DEPs) were identified using an abundance ratio threshold (>1.5) together with an adjusted *p*-value <0.05. Statistical significance was assessed using the background-based *t* test implemented in Proteome Discoverer 2.5, and *p*-values were adjusted for multiple testing using the Benjamini–Hochberg false discovery rate (FDR) correction. Protein and peptide identifications were validated using the Percolator algorithm with a target-decoy approach, applying a 1% FDR. Each condition was analyzed in triplicate, with periodic quality control injections to ensure data reproducibility. Bioinformatics analyses, including Gene Ontology (GO), Reactome pathway enrichment, and protein–protein interaction networks, were conducted using STRING and ClueGO, applying corrected *p*-value thresholds (<0.05).

To assess clinical relevance, we queried the independent primary and metastatic breast cancer cohorts via cBioPortal, evaluating genomic amplification of genes encoding downregulated proteins in both Alovudine-treated and mtDNA-depleted cells. Amplification frequencies were analyzed separately for each cohort and interpreted descriptively because of differences in cohort composition and molecular characteristics. Fisher’s exact test was performed for each metastatic-versus-primary comparison. This integrative approach enabled the identification of recurrently amplified genes associated with proteins downregulated following Alovudine treatment and mtDNA depletion.

#### Origin of Cell lines

Parental MDA-MB-231 (HTB-26) and MCF-7 (HTB-22) were obtained from the American Type Culture Collection. Cells were grown at 37 °C in a 5% CO2 humidified incubator. Cells were cultured in Dulbecco’s Modified Eagle Medium (DMEM; Sigma-Aldrich, Darmstadt, Germany, #D6546), supplemented with 10% Fetal Bovine Serum, 2 mM GlutaMAX, and 1% Penicillin-Streptomycin. When cells reached approximately 70 to 80% confluency, they were trypsinized, harvested, washed and used for experiments or passaged for further expansion.

#### Alovudine-Treatment of MDA-MB-231 Cells

Alovudine-related shotgun proteomics experiments were performed after a 6-days pre-treatment of MDA-MB-231 cells, as we previously described [6]. More specifically, 250,000 cells were plated in T25 flasks and incubated for 6 days with Alovudine, at a concentration of 1 μM. Cells were treated at day 1 and media was renewed with Alovudine at day 4. Control cells processed in parallel received vehicle alone.

#### Generating mtDNA-depleted Cells

mtDNA-depleted MCF-7 and MDA-MB-231 cells were generated using standard protocols, by serially culturing the cells with ethidium bromide (EtBr), essentially as previously described (7, 8). Briefly, cells were treated with EtBr at 50 ng/ml for 2 weeks, followed by 25 ng/ml for an additional 2 weeks, and subsequently maintained at 12.5 ng/ml. Throughout the entire procedure, cells were cultured in medium supplemented with uridine (50 μg/ml) to support viability and proliferation in the absence of mitochondrial DNA, as is standard practice for mtDNA-depleted cells. For downstream proteomic analyses, equal amounts of total protein were used for each sample to account for potential differences in cell growth between mtDNA-depleted and parental cells.

#### Seahorse Metabolic Flux Analysis

To evaluate real-time Oxygen Consumption Rates (OCRs) and Extracellular Acidification Rates (ECARs), we used the Seahorse Extracellular Flux (XFe96) analyzer (Agilent/Seahorse Bioscience). Approximately, 30,000 cells per well were seeded in the XFe-96-well plates and incubated in a humidified atmosphere at 37 °C and 5% CO_2_ for 24 h. Cells were incubated in 175 μl/well of XF assay media at 37 °C, in a non-CO2 incubator for 1 h before the Seahorse assay. The XFe-96 sensor cartridge was loaded with 25 μl of 80 mM glucose, 9 μM oligomycin and 1 M 2-deoxyglucose (for ECAR measurements) or 10 μM oligomycin, 10 μM FCCP, 10 μM rotenone and 10 μM antimycin A (for OCR measurements) in XF assay media. Measurements were normalized by Hoechst staining. Data sets were analyzed using Agilent Seahorse Wave software (https://www.agilent.com/en/support/seahorse-xf-software-releases?srsltid=AfmBOorWd3Nodqyj3HMwhtPd9Jqpr-4BLK18mnBk8bsnhydpMf13pFY9).

#### Quantification of mtDNA by qPCR

DNA extractions were performed using the Monarch Genomic DNA Purification Kit (New England Biolabs, #T3010S) from cell pellets of 1 × 10^6^ cells. DNA was subsequently diluted to a final concentration of 5 ng/μl. Relative mitochondrial DNA copy number was obtained by using the Relative Human Mitochondrial DNA Copy Number Quantification qPCR Assay Kit (ScienCell, California, USA, #8938) which quantifies mtDNA levels by normalizing them to a single-copy nuclear genomic region located on chromosome 17. Briefly, a 10 μl reaction contained: 5 μl Reaction MasterMix, 1 μl primers (10 μM, Fwd + Rv mixed), 1 μl of DNA (5 ng) and 3 μl water. qPCR reactions were performed in duplicate or triplicate, and three biological repeats were analyzed for each condition. qPCRs were performed in a StepOnePlus Real-Time PCR System (Applied Biosystems), with conditions as follows: 95 °C for 10 min, followed by 32 cycles of 95 °C for 20 s, 52 °C for 20 s, and 72 °C for 45 s. The relative mtDNA levels were calculated using the ΔΔCT method.

#### Colony Formation Assay

Single-cell suspensions were seeded in 6-well tissue culture plates at a density of 25 cells/cm2. Cells were cultured in DMEM (Sigma-Aldrich, Darmstadt, Germany, #D6546) supplemented with 10% FBS, 1X GlutaMAX and 1% Penicillin–Streptomycin. Cells were grown for 14 days and maintained at 37 °C in a humidified air incubator containing carbon dioxide (5%). Media were replaced once after the first 7 days with fresh media. After 14 days, colonies were fixed with 100% methanol at 4 °C for 30 min and stained with 0.5% Crystal Violet for 10 min at RT. Stained cell colonies in each well were counted, and the results were representative of three independent experiments.

#### Protein extraction and sample preparation

Protein extracts were prepared from MDA-MB-231 cells, either untreated or treated with Alovudine, as well as from mtDNA-depleted MDA-MB-231 cells and their parental controls, and from mtDNA-depleted MCF-7 cells and corresponding controls. Sample preparation was performed using the EasyPep Mini MS Sample Preparation Kit (Thermo Fisher Scientific), following the manufacturer’s protocol. Briefly, approximately 1 × 10^6^ cells per well were lysed in buffer containing Universal Nuclease by 10 cycles of pipetting. Total protein concentration was determined using the Microplate BCA Protein Assay Kit (Pierce, Thermo Fisher Scientific). A 30 μg aliquot of protein was reduced and alkylated in 50 μl of reagent solution at 95 °C for 10 min, followed by enzymatic digestion with 50 μl of trypsin/Lys-C at 37 °C for 3 h. Peptides were desalted using cleanup columns, dried under vacuum, and resuspended in 0.1% TFA for LC-MS/MS analysis. Four biological replicates were processed for each experimental group.

#### Mass spectrometry-based quantitative proteomics

LC-MS/MS was performed using an Ultimate 3000 nano-UHPLC system coupled to an Orbitrap Fusion Lumos Tribrid mass spectrometer (Thermo Fisher Scientific), as previously described (9), with minor modifications. Peptide separation (1.2 μg per sample) was carried out on a reverse-phase C18 analytical column (2 μm, 500 × 0.075 mm) using a linear gradient of 4 to 50% acetonitrile over 120 min at a constant flow rate of 300 nl/min on an EASY-nLC 1000 system (Thermo Fisher Scientific, Waltham, MA). Eluted peptides were ionized and introduced into the Orbitrap Fusion. MS1 scans were acquired in the Orbitrap at a resolution of 120,000 (at m/z 200) over a mass range of m/z 375–1500. Data-dependent MS/MS was triggered for precursor ions exceeding an intensity threshold of 3.0 × 10^4^. Precursors were isolated using a 4.0 Da window and fragmented by higher-energy collisional dissociation with normalized collision energy of 30%. Fragment ions were detected in the Orbitrap at a resolution of 50,000 (at m/z 200). Dynamic exclusion was set to 45 s. Each condition was analyzed in triplicate. To monitor data quality, blank samples (30% acetonitrile in water) and a quality control sample consisting of HeLa lysate digest were injected every four runs.

#### Proteomics data processing and bioinformatics analysis

Raw MS data files were analyzed using Proteome Discoverer software (version 2.5, Thermo Fisher Scientific; https://www.thermofisher.com/it/en/home/industrial/mass-spectrometry/liquid-chromatography-mass-spectrometry-lc-ms/lc-ms-software/multi-omics-data-analysis/proteome-discoverer-software.html), applying mass accuracy thresholds of 10 ppm for precursor ions (MS1) and 0.02 Da for fragment ions (MS2). Spectral data were matched against the *Homo sapiens* Swiss-Prot reviewed UniProtKB database (TaxID 9606), comprising a total of 20,432 curated protein sequences. Database searching was performed using the following search criteria: enzymatic digestion by trypsin allowing up to two missed cleavage sites; carbamidomethylation of cysteine residues as a static modification; and oxidation of methionine, histidine, and tryptophan, along with N-terminal acetylation, as dynamic modifications. Protein and peptide identifications were validated using the Percolator algorithm with a target-decoy approach, applying a FDR cut-off set at 1%. Quantification was performed in a label-free manner, requiring a minimum of two unique peptides per protein. Label-free quantification was carried out using the Minora Feature Detector in the processing workflow, followed by the Feature Mapper and Precursor Ions Quantifier nodes in the consensus workflow, all operated with default parameters. Relative abundance was calculated through pairwise ratio comparisons, and differential protein abundance between experimental groups was assessed using the Background-Based *t* test implemented in Proteome Discoverer for nested study designs. To account for multiple hypotheses testing, *p*-values were adjusted using the Benjamini–Hochberg FDR correction implemented in Proteome Discoverer, and the resulting adjusted *p*-values were used for all downstream analyses, including volcano plot generation. The mass spectrometry proteomics data have been deposited to the ProteomeXchange Consortium via the PRIDE (10) partner repository with the dataset identifier PXD077655. The ratio- and adjusted p-value-based filtering technique was applied to select the DEPs with high confidence (abundance ratio >1.5; adjusted *p*-value <0.05). Volcano plots were generated using log_2_ (fold change) and −log_10_(adjusted *p*-value). Proteins were annotated using MitoCarta3.0 (1136 curated mitochondrial proteins) and grouped as OXPHOS (n = 169), non-OXPHOS mitochondrial, or other proteins.

Bioinformatics analysis was performed on the identified lists of DEPs to assess GO terms, pathway enrichment, and protein–protein interaction (PPI) networks using the STRING v12.0 platform (http://string-db.org). STRING enrichment analysis was performed using *H. sapiens* as taxonomy in order to identify significantly enriched GO Biological Process, as well as Reactome and KEGG pathways. For each experimental comparison, the statistical background was restricted to the proteins identified in the corresponding LC-MS/MS experiment, rather than the complete human proteome. This approach avoids enrichment bias associated with using the complete reference proteome and provides a more appropriate statistical framework for quantitative proteomic datasets. Enrichment significance was calculated using the hypergeometric test implemented in STRING, and *p*-values were corrected for multiple testing using the Benjamini–Hochberg FDR procedure. The enrichment output included the observed gene count, background gene count, enrichment strength, FDR-adjusted *p*-values, and the proteins associated with each enriched term. Functional enrichment was also independently performed using ClueGO, a plugin for Cytoscape v3.10.2 (http://www.ici.upmc.fr/cluego). ClueGO was used to identify significantly enriched Reactome and KEGG pathways from the DEP lists using a right-sided hypergeometric test with Benjamini–Hochberg correction for multiple testing and to organize functionally related pathways into biologically meaningful networks based on shared proteins. Only pathways with corrected *p*-values <0.05 were considered significant. Because ClueGO was employed primarily for network organization and visualization rather than statistical interpretation, analyses were performed using the default *H. sapiens* reference set.

### Genomic Alteration Analysis Across Metastatic and Primary Breast Cancer Cohorts

To investigate whether genes encoding proteins downregulated following Alovudine treatment and/or mtDNA depletion exhibited recurrent copy-number amplification in breast cancer, genomic alteration frequencies were retrieved from the cBioPortal platform (https://www.cbioportal.org).

Metastatic tumors were represented by two independent cohorts: the Metastatic Breast Cancer (MBC) Project (11) (Provisional, December 2021; sequencing data from n = 379 samples from 301 patients with clinically confirmed metastatic disease) and the Institut National de la Santé et de la Recherche Médicale (INSERM) Metastatic Breast Cancer study (12) (n = 216 samples from 216 patients). Primary tumors were represented by The Cancer Genome Atlas (TCGA) Breast Invasive Carcinoma (https://datacatalog.mskcc.org/dataset/10408) (n = 1084 samples from 1084 patients) and CPTAC Breast Cancer (13) (n = 122 samples from 122 patients). For each gene of interest, we extracted the frequency and type of genomic alterations. To explore whether genes encoding downregulated proteins showed recurrent copy-number amplification across breast cancer cohorts, amplification frequencies were extracted separately for each dataset. Because the cohorts differ in sample composition, subtype distribution, and copy-number calling procedures, dataset-specific frequencies were interpreted descriptively rather than as definitive evidence of metastatic specificity.

To evaluate the consistency of amplification patterns across independent cohorts, pairwise two-tailed Fisher's exact tests were performed for each metastatic-versus-primary comparison (MBC Project vs TCGA, MBC Project vs CPTAC, INSERM vs TCGA, INSERM vs CPTAC). Statistical significance was defined as *p* < 0.05. All analyses were conducted using default cBioPortal parameters, and dataset identifiers and accession links are provided in the Supplementary Information.

## Results

### Overview of the Experimental Strategy

**Fig. 1:**
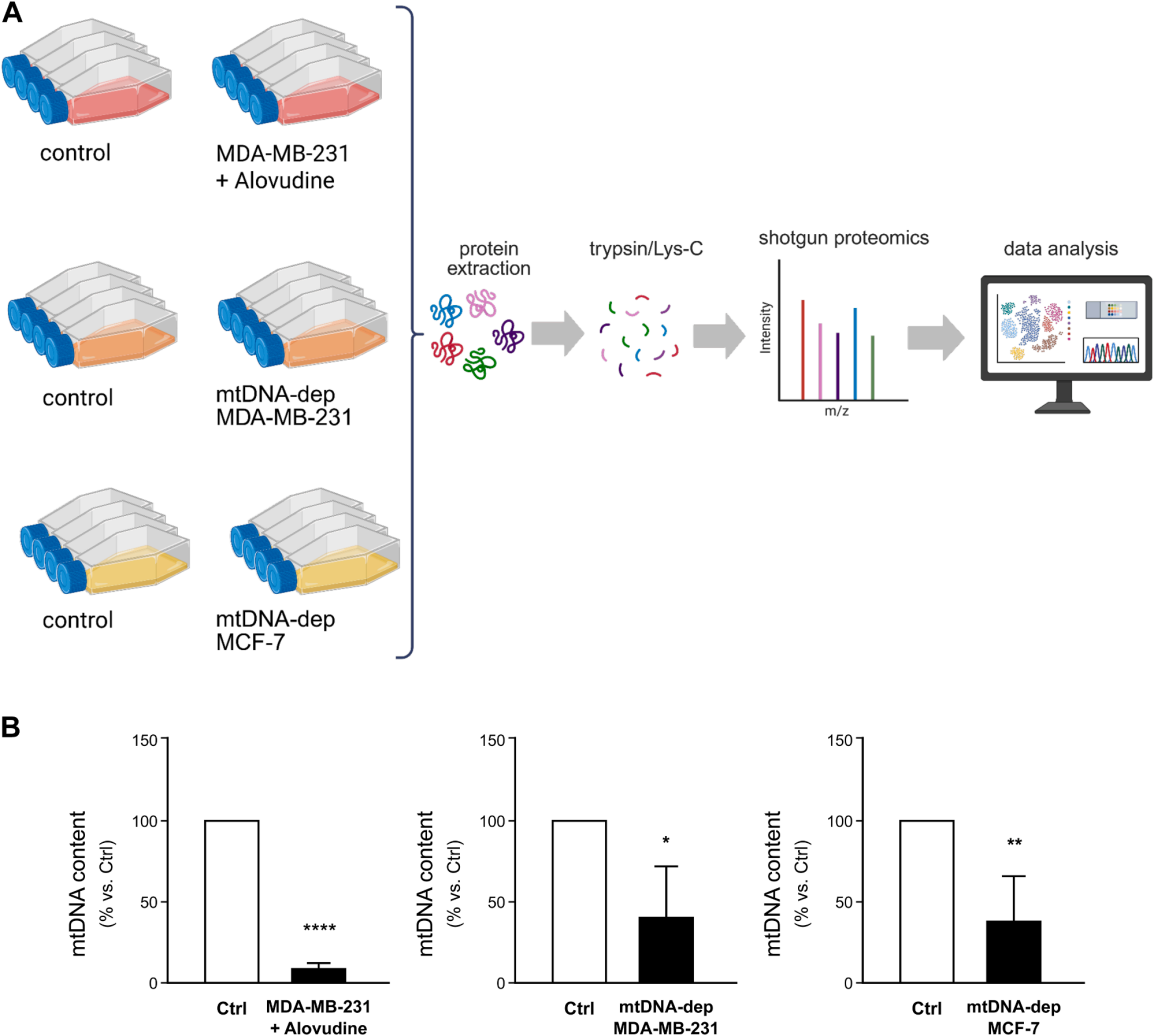
**Experimental workflow.***A,* a schematic diagram showing the workflow of proteomic analysis. The experiment included three comparisons: Alovudine-treated MDA-MB-231 cells *versus* control, mtDNA-depleted MDA-MB-231 cells *versus* control, as well as mtDNA-depleted MCF-7 cells *versus* control. *B,* mtDNA quantification by qPCR to assess mitochondrial content. Relative mitochondrial DNA copy number was obtained using a Relative Human Mitochondrial DNA Copy Number Quantification qPCR Assay Kit. Data are shown as the mean ± SD (n = 3). mtDNA, mitochondrial DNA.

To investigate the molecular consequences of mtDNA depletion and its impact on metastatic potential in breast cancer cells, we employed a comparative proteomics strategy (Fig. 1*A*) using three conditions: MDA-MB-231 cells treated with Alovudine, mtDNA-depleted MDA-MB-231 cells generated with ethidium bromide, and mtDNA-depleted MCF-7 cells as an additional mtDNA deficient model (Fig. 1*B*). This design allowed us to distinguish drug specific effects from general outcomes of mitochondrial loss. The mtDNA-depleted cells were first functionally validated by Seahorse metabolic flux analysis, confirming loss of mitochondrial respiration and compensatory increases in glycolysis. Following this validation, the comparative proteomics experiment was performed.

### Functional Characterization of mtDNA-Depleted Cells Deficient in Mitochondrial Activity

We already demonstrated that Alovudine, an established mtDNA synthesis inhibitor, induces a metabolic shift towards glycolysis and prevents spontaneous cancer cell metastasis, without significant toxicity (6). Here, we characterize the mitochondrial-deficient models, mtDNA-depleted MDA-MB-231 and MCF-7 cells, mitochondria-depleted derivatives that lack mitochondrial DNA and are therefore unable to perform OXPHOS, by Seahorse analysis (Supplemental Fig. S1). As expected, mtDNA-depleted MDA-MB-231 cells exhibited a complete loss of OCR, accompanied by marked reductions in basal respiration, mitochondrial ATP production, and maximal respiration (Supplemental Fig. S1*A*). These defects were paralleled by compensatory increases in ECAR, reflecting enhanced glycolysis, but with diminished glycolytic reserve and reserve capacity (Supplemental Fig. S1*B*). Consistent with their mitochondrial deficiency, mtDNA-depleted MDA-MB-231 cells showed significant reductions in mtDNA content and impaired colony formation (Supplemental Fig. S1*C*). Similarly, mtDNA-depleted MCF-7 cells (Supplemental Fig. S2) displayed a complete loss of OCR, with profound decreases in basal respiration, mitochondrial ATP production, and maximal respiration (Supplemental Figure S2*A*). These cells also demonstrated compensatory increases in ECAR, associated with elevated glycolysis (Supplemental Fig. S2*B*). In line with their mitochondrial dysfunction, mtDNA-depleted MCF-7 cells exhibited significant reductions in mtDNA levels and colony-forming ability (Supplemental Figure S2*C*).

### Global Proteomic Changes upon mtDNA Synthesis Inhibition

To gain deeper insight into cellular responses to inhibited mtDNA synthesis, we performed a proteomic analysis of MDA-MB-231 cells treated with Alovudine, as well as of mtDNA-depleted MDA-MB-231 and MCF-7 cells alongside their respective controls. Four independent biological replicates for each sample were analyzed using high-throughput shotgun proteomics.

**Fig. 2:**
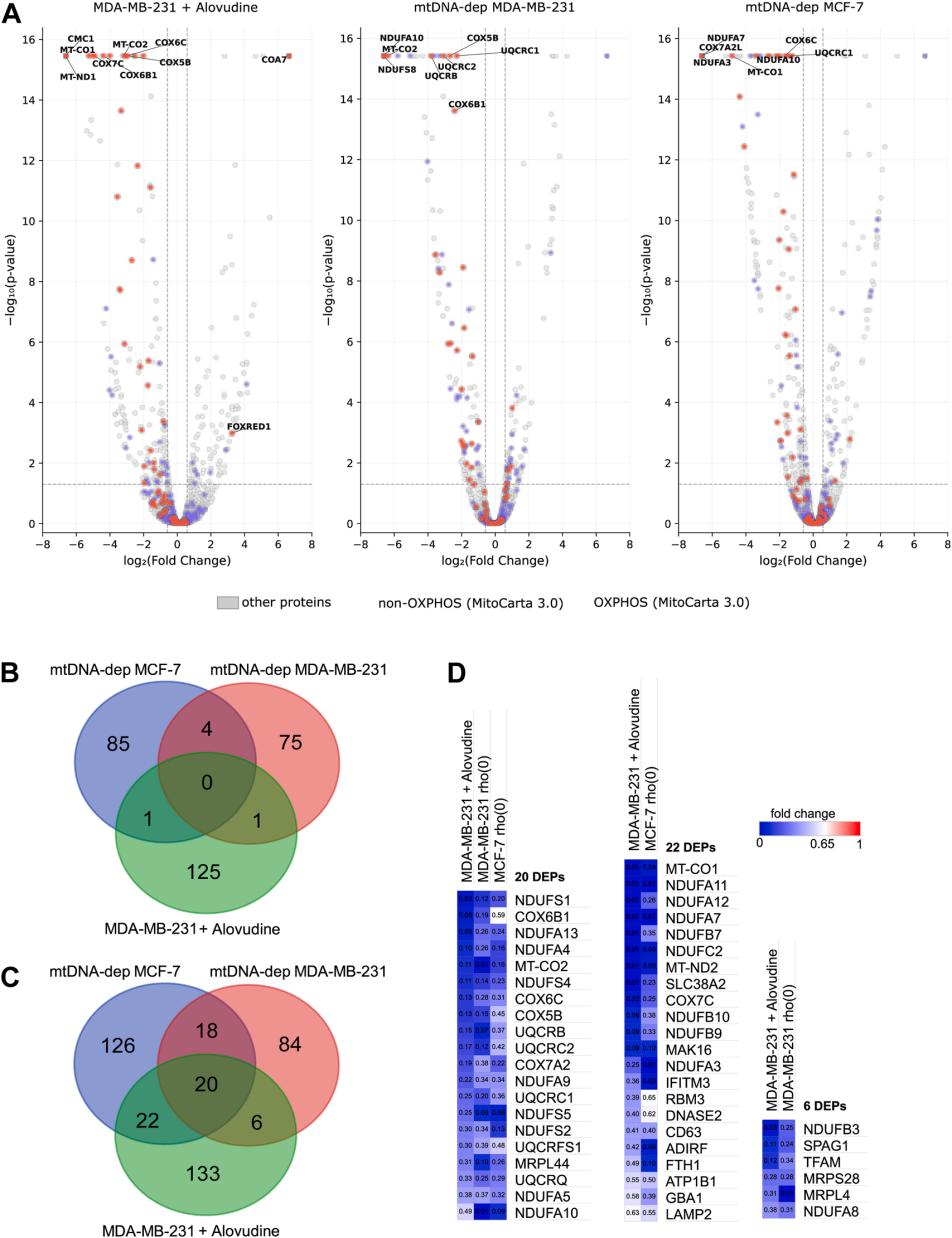
**Proteomics results.***A,* Volcano plots showing the global proteome changes in MDA-MB-231 cells treated by Alovudine *versus* control, as well as in mtDNA-depleted MDA-MB-231 and MCF-7 cells *versus* control cells. Individual proteins are represented by points, with oxidative phosphorylation and non-oxidative phosphorylation proteins highlighted according to MitoCarta3.0 annotation to distinguish them from other non-mitochondrial proteins. *B and C,* Venn diagrams representing the overlap among upregulated (on the *left*) and downregulated (on the *right*) proteins identified by LC-MS/MS analysis. *D,* Heatmaps representing the commonly downregulated DEPs across the three samples. The color key is proportional to the fold change. Proteins in the three heatmaps are arranged in descending order based on their level of downregulation in MDA-MB-231 cells treated with Alovudine. DEP, differentially expressed protein; mtDNA, mitochondrial DNA.

Proteomic analysis identified 309 DEPs between untreated and Alovudine-treated cells, with 128 upregulated and 181 downregulated following treatment. In addition, 208 and 276 DEPs were detected in mtDNA-depleted cells derived from MDA-MB-231 and MCF-7 lines, respectively (Supplemental Table S1). The DEPs are visualized in volcano plots and ranked according to fold change values greater than 1.5, with all selected proteins meeting the statistical significance threshold of *p* < 0.05 (Fig. 2*A*). To assess the functional identity of DEPs, all volcano plots were overlaid with MitoCarta3.0 annotation, distinguishing OXPHOS from other non-OXPHOS mitochondrial proteins and the remaining proteome. In all three experimental conditions, OXPHOS proteins (shown in red) were markedly enriched among the most significantly downregulated DEPs, with several subunits reaching the maximum detectable significance threshold (adjusted *p*-value ≤10^−15^). The DEPs in Alovudine-treated cells identified as mitochondrial proteins are all reported in Table 1. Notably, the overlap between up- (Fig. 2*B*) and downregulated (Fig. 2*C*) proteins in Alovudine-treated cells and those in mtDNA-depleted cells revealed that 51 proteins modulated by Alovudine were also altered in mtDNA-depleted cells, suggesting their regulation is directly influenced by mitochondrial DNA. Among these, 20 proteins were consistently downregulated following Alovudine treatment and in both mtDNA-depleted cell lines; 22 were downregulated in Alovudine-treated cells and mtDNA-depleted MCF-7 cells; and six were downregulated in Alovudine-treated cells and mtDNA-depleted MDA-MB-231 cells (Fig. 2*D* and Table 2).

**Table 1:** Mitochondrial Proteins Modulated by Alovudine Treatment

Accession	Description	Gene Symbol	Abundance ratio: MDA-MB-231 + Alovudine/CTRL	Abundance Ratio Adj. *p*-value
Mitochondrial ribosomes and genetic system				
Q9BYN8	28S ribosomal protein S26, mitochondrial	MRPS26	0.14	0.00
Q9Y2Q9	28S ribosomal protein S28, mitochondrial	MRPS28	0.28	0.04
Q13084	39S ribosomal protein L28, mitochondrial	MRPL28	0.07	0.00
Q9BYD3	39S ribosomal protein L4, mitochondrial	MRPL4	0.31	0.01
Q9H9J2	39S ribosomal protein L44, mitochondrial	MRPL44	0.31	0.05
P21589-1	5′-nucleotidase ∗	NT5E	0.66	0.04
P27144	Adenylate kinase 4, mitochondrial	AK4	0.66	0.01
P33316-3	Deoxyuridine 5′-triphosphate nucleotidohydrolase, mitochondrial ∗	DUT	2.03	0.05
Q14566	DNA replication licensing factor MCM6 ∗	MCM6	1.96	0.01
P29372	DNA-3-methyladenine glycosylase ∗	MPG	11.43	0.00
P39748	Flap endonuclease 1	FEN1	1.93	0.00
O00142	Thymidine kinase 2, mitochondrial	TK2	0.07	0.00
Q00059	Transcription factor A, mitochondrial	TFAM	0.12	0.00
Oxidative phosphorylation				
Q7Z7K0	COX assembly mitochondrial protein homolog	CMC1	0.01	0.00
P31930	Cytochrome b-c1 complex subunit 1, mitochondrial	UQCRC1	0.25	0.00
P22695	Cytochrome b-c1 complex subunit 2, mitochondrial	UQCRC2	0.17	0.00
P14927	Cytochrome b-c1 complex subunit 7	UQCRB	0.15	0.00
O14949	Cytochrome b-c1 complex subunit 8	UQCRQ	0.33	0.00
P47985	Cytochrome b-c1 complex subunit Rieske, mitochondrial	UQCRFS1	0.30	0.00
Q96BR5	Cytochrome c oxidase assembly factor 7	COA7	100.00	0.00
P00395	Cytochrome c oxidase subunit 1	MT-CO1	0.01	0.00
P00403	Cytochrome c oxidase subunit 2	MT-CO2	0.11	0.00
P13073	Cytochrome c oxidase subunit 4 isoform 1, mitochondrial	COX4I1	0.33	0.00
P20674	Cytochrome c oxidase subunit 5A, mitochondrial	COX5A	0.56	0.00
P10606	Cytochrome c oxidase subunit 5B, mitochondrial	COX5B	0.13	0.00
P14854	Cytochrome c oxidase subunit 6B1	COX6B1	0.05	0.00
P09669	Cytochrome c oxidase subunit 6C	COX6C	0.13	0.00
P14406	Cytochrome c oxidase subunit 7A2, mitochondrial	COX7A2	0.19	0.00
P15954	Cytochrome c oxidase subunit 7C, mitochondrial	COX7C	0.03	0.00
O00483	Cytochrome c oxidase subunit NDUFA4	NDUFA4	0.10	0.00
Q96CU9	FAD-dependent oxidoreductase domain-containing protein 1	FOXRED1	9.57	0.00
O95299	NADH dehydrogenase [ubiquinone] 1 alpha subcomplex subunit 10, mitochondrial	NDUFA10	0.49	0.02
Q86Y39-1	NADH dehydrogenase [ubiquinone] 1 alpha subcomplex subunit 11	NDUFA11	0.01	0.00
Q9UI09	NADH dehydrogenase [ubiquinone] 1 alpha subcomplex subunit 12	NDUFA12	0.01	0.00
Q9P0J0	NADH dehydrogenase [ubiquinone] 1 alpha subcomplex subunit 13	NDUFA13	0.06	0.00
O95167	NADH dehydrogenase [ubiquinone] 1 alpha subcomplex subunit 3	NDUFA3	0.25	0.05
Q16718	NADH dehydrogenase [ubiquinone] 1 alpha subcomplex subunit 5	NDUFA5	0.38	0.01
O95182	NADH dehydrogenase [ubiquinone] 1 alpha subcomplex subunit 7	NDUFA7	0.01	0.00
P51970	NADH dehydrogenase [ubiquinone] 1 alpha subcomplex subunit 8	NDUFA8	0.38	0.02
Q16795	NADH dehydrogenase [ubiquinone] 1 alpha subcomplex subunit 9, mitochondrial	NDUFA9	0.22	0.00
O96000	NADH dehydrogenase [ubiquinone] 1 beta subcomplex subunit 10	NDUFB10	0.08	0.00
Q9NX14-1	NADH dehydrogenase [ubiquinone] 1 beta subcomplex subunit 11, mitochondrial	NDUFB11	0.10	0.00
O43676	NADH dehydrogenase [ubiquinone] 1 beta subcomplex subunit 3	NDUFB3	0.03	0.00
O95168-1	NADH dehydrogenase [ubiquinone] 1 beta subcomplex subunit 4	NDUFB4	0.23	0.00
P17568	NADH dehydrogenase [ubiquinone] 1 beta subcomplex subunit 7	NDUFB7	0.01	0.00
O95169	NADH dehydrogenase [ubiquinone] 1 beta subcomplex subunit 8, mitochondrial	NDUFB8	0.01	0.00
Q9Y6M9	NADH dehydrogenase [ubiquinone] 1 beta subcomplex subunit 9	NDUFB9	0.09	0.00
O95298-1	NADH dehydrogenase [ubiquinone] 1 subunit C2	NDUFC2	0.01	0.00
O75306-1	NADH dehydrogenase [ubiquinone] iron-sulfur protein 2, mitochondrial	NDUFS2	0.30	0.00
O43181	NADH dehydrogenase [ubiquinone] iron-sulfur protein 4, mitochondrial	NDUFS4	0.11	0.00
O43920	NADH dehydrogenase [ubiquinone] iron-sulfur protein 5	NDUFS5	0.25	0.01
P28331-1	NADH-ubiquinone oxidoreductase 75 kDa subunit, mitochondrial	NDUFS1	0.03	0.00
P03886	NADH-ubiquinone oxidoreductase chain 1	MT-ND1	0.01	0.00
P03891	NADH-ubiquinone oxidoreductase chain 2	MT-ND2	0.01	0.00
P03905	NADH-ubiquinone oxidoreductase chain 4	MT-ND4	0.01	0.00
P03915	NADH-ubiquinone oxidoreductase chain 5	MT-ND5	0.01	0.00
Q8N573-1	Oxidation resistance protein 1	OXR1	17.29	0.00
P04179	Superoxide dismutase [Mn], mitochondrial	SOD2	0.37	0.00
Q9H3M7	Thioredoxin-interacting protein ∗	TXNIP	0.08	0.00
Q13488	V-type proton ATPase 116 kDa subunit a isoform 3 ∗	TCIRG1	0.17	0.00
TCA cycle and intermediary metabolism				
P12694	2-oxoisovalerate dehydrogenase subunit alpha, mitochondrial	BCKDHA	0.06	0.00
Q96CM8	Acyl-CoA synthetase family member 2, mitochondrial	ACSF2	0.56	0.02
Q15847	Adipogenesis regulatory factor ∗	ADIRF	0.42	0.00
Q13011	Delta (3, 5)-Delta (2, 4)-dienoyl-CoA isomerase, mitochondrial	ECH1	0.62	0.01
P51648	Fatty aldehyde dehydrogenase ∗	ALDH3A2	0.42	0.04
P02794	Ferritin heavy chain ∗	FTH1	0.49	0.00
P09972	Fructose-bisphosphate aldolase C ∗	ALDOC	0.61	0.00
Q9UMS0	NFU1 iron-sulfur cluster scaffold homolog, mitochondrial	NFU1	3.88	0.02
P04181-1	Ornithine aminotransferase, mitochondrial	OAT	1.86	0.01
Mitochondrial transporters				
Q99543-1	DNAJ homolog subfamily C member 2 ∗	DNAJC2	11.66	0.00
Q8WXX5	DNAJ homolog subfamily C member 9 ∗	DNAJC9	2.05	0.02
Q9UBX3	Mitochondrial dicarboxylate carrier	SLC25A10	2.07	0.02
Q96QD8	Sodium-coupled neutral amino acid transporter 2 ∗	SLC38A2	0.01	0.00
Q9UP95	Solute carrier family 12 member 4 ∗	SLC12A4	0.08	0.00
Q8NBI5	Solute carrier family 43 member 3 ∗	SLC43A3	0.40	0.05
Mitochondrial morphology and dynamics, mitophagy, apoptosis				
O95994	Anterior gradient protein 2 homolog ∗	AGR2	0.32	0.00
Q9BRQ8-1	Apoptosis-inducing factor 2	AIFM2	0.47	0.00
P35869	Aryl hydrocarbon receptor ∗	AHR	0.30	0.01
P55287-1	Cadherin-11 ∗	CDH11	0.29	0.00
Q9NR28-1	Diablo homolog, mitochondrial	DIABLO	0.66	0.04
P04062	Glucosylceramidase ∗	GBA1	0.58	0.01
Q96HS1-1	Serine/threonine-protein phosphatase Pgam5, mitochondrial	PGAM5	2.25	0.02
Q9UJZ1	Stomatin-like protein 2, mitochondrial	STOML2	0.65	0.02
Protein import machinery				
Q9NP58	ATP-binding cassette sub-family B member 6, mitochondrial	ABCB6	0.59	0.00
O95757	Heat shock 70 kDa protein 4L	HSPA4L	2.02	0.01
Other mitochondrial functions (ADP-ribosylation)				
Q9NX46	Poly(ADP-ribose) glycohydrolase ARH3	ADPRS	0.55	0.01

Differentially expressed proteins marked with an asterisk (∗) are those that, based on the Subcellular Locations tool from COMPARTMENTS (https://compartments.jensenlab.org/Search), are also found in the mitochondria, but are more confidently located in other intracellular compartments (such as the cytosol, nucleus, plasma membrane, etc.).

**Table 2:** Downregulated Proteins Common Between MDA-MB-231 Cells Treated with Alovudine and mtDNA-depleted Cells

Accession	Description	Gene Symbol	Abundance Ratio: MDA-MB-231 + Alovudine/CTRL	Abundance Ratio: mtDNA-dep MDA-MB-231/CTRL	Abundance Ratio: mtDNA-dep MCF-7/CTRL
MDA-MB-231 + Alovudine & mitochondrial DNA(mtDNA)-dep MDA-MB-231 & mtDNA-dep MCF-7:					
Q9H9J2	39S ribosomal protein L44, mitochondrial	MRPL44	0.31	0.1	0.26
P31930	Cytochrome b-c1 complex subunit 1, mitochondrial	UQCRC1	0.25	0.20	0.36
P22695	Cytochrome b-c1 complex subunit 2, mitochondrial	UQCRC2	0.17	0.12	0.42
P14927	Cytochrome b-c1 complex subunit 7	UQCRB	0.15	0.07	0.37
O14949	Cytochrome b-c1 complex subunit 8	UQCRQ	0.33	0.25	0.29
P47985	Cytochrome b-c1 complex subunit Rieske, mitochondrial	UQCRFS1	0.30	0.39	0.48
P00403	Cytochrome c oxidase subunit 2	MT-CO2	0.11	0.01	0.16
P10606	Cytochrome c oxidase subunit 5B, mitochondrial	COX5B	0.13	0.15	0.45
P14854	Cytochrome c oxidase subunit 6B1	COX6B1	0.05	0.19	0.59
P09669	Cytochrome c oxidase subunit 6C	COX6C	0.13	0.28	0.32
P14406	Cytochrome c oxidase subunit 7A2, mitochondrial	COX7A2	0.19	0.38	0.22
O00483	Cytochrome c oxidase subunit NDUFA4	NDUFA4	0.1	0.27	0.16
O95299	NADH dehydrogenase [ubiquinone] 1 alpha subcomplex subunit 10, mitochondrial	NDUFA10	0.49	0.01	0.09
Q9P0J0	NADH dehydrogenase [ubiquinone] 1 alpha subcomplex subunit 13	NDUFA13	0.06	0.26	0.24
Q16718	NADH dehydrogenase [ubiquinone] 1 alpha subcomplex subunit 5	NDUFA5	0.38	0.37	0.32
Q16795	NADH dehydrogenase [ubiquinone] 1 alpha subcomplex subunit 9, mitochondrial	NDUFA9	0.22	0.34	0.34
O75306-1	NADH dehydrogenase [ubiquinone] iron-sulfur protein 2, mitochondrial	NDUFS2	0.3	0.34	0.13
O43181	NADH dehydrogenase [ubiquinone] iron-sulfur protein 4, mitochondrial	NDUFS4	0.11	0.14	0.23
O43920	NADH dehydrogenase [ubiquinone] iron-sulfur protein 5	NDUFS5	0.25	0.08	0.06
P28331-1	NADH-ubiquinone oxidoreductase 75 kDa subunit, mitochondrial	NDUFS1	0.03	0.12	0.20
MDA-MB-231 + Alovudine & mtDNA-dep MDA-MB-231:					
Q9Y2Q9	28S ribosomal protein S28, mitochondrial	MRPS28	0.28	0.28	
Q9BYD3	39S ribosomal protein L4, mitochondrial	MRPL4	0.31	0.01	
P51970	NADH dehydrogenase [ubiquinone] 1 alpha subcomplex subunit 8	NDUFA8	0.38	0.31	
O43676	NADH dehydrogenase [ubiquinone] 1 beta subcomplex subunit 3	NDUFB3	0.03	0.25	
Q07617	Sperm-associated antigen 1	SPAG1	0.11	0.24	
Q00059	Transcription factor A, mitochondrial	TFAM	0.12	0.34	
MDA-MB-231 + Alovudine & mtDNA-dep MCF-7:					
Q15847	Adipogenesis regulatory factor	ADIRF	0.42		0.06
P08962	CD63 antigen	CD63	0.41		0.40
P00395	Cytochrome c oxidase subunit 1	MT-CO1	0.01		0.04
P15954	Cytochrome c oxidase subunit 7C, mitochondrial	COX7C	0.03		0.25
O00115	Deoxyribonuclease-2-alpha	DNASE2	0.40		0.62
P02794	Ferritin heavy chain	FTH1	0.49		0.10
P04062	Glucosylceramidase	GBA1	0.58		0.39
Q01628	Interferon-induced transmembrane protein 3	IFITM3	0.36		0.03
P13473-1	Lysosome-associated membrane glycoprotein 2	LAMP2	0.63		0.55
Q86Y39-1	NADH dehydrogenase [ubiquinone] 1 alpha subcomplex subunit 11	NDUFA11	0.01		0.01
Q9UI09	NADH dehydrogenase [ubiquinone] 1 alpha subcomplex subunit 12	NDUFA12	0.01		0.26
O95167	NADH dehydrogenase [ubiquinone] 1 alpha subcomplex subunit 3	NDUFA3	0.25		0.01
O95182	NADH dehydrogenase [ubiquinone] 1 alpha subcomplex subunit 7	NDUFA7	0.01		0.01
O96000	NADH dehydrogenase [ubiquinone] 1 beta subcomplex subunit 10	NDUFB10	0.08		0.38
P17568	NADH dehydrogenase [ubiquinone] 1 beta subcomplex subunit 7	NDUFB7	0.01		0.35
Q9Y6M9	NADH dehydrogenase [ubiquinone] 1 beta subcomplex subunit 9	NDUFB9	0.09		0.33
O95298-1	NADH dehydrogenase [ubiquinone] 1 subunit C2	NDUFC2	0.01		0.04
P03891	NADH-ubiquinone oxidoreductase chain 2	MT-ND2	0.01		0.05
Q9BXY0	Protein MAK16 homolog	MAK16	0.09		0.10
P98179	RNA-binding protein 3	RBM3	0.39		0.65
P05026	Sodium/potassium-transporting ATPase subunit beta-1	ATP1B1	0.55		0.50
Q96QD8	Sodium-coupled neutral amino acid transporter 2	SLC38A2	0.01		0.23

### Mitochondrial Responses Across mtDNA Depletion Models

**Fig. 3:**
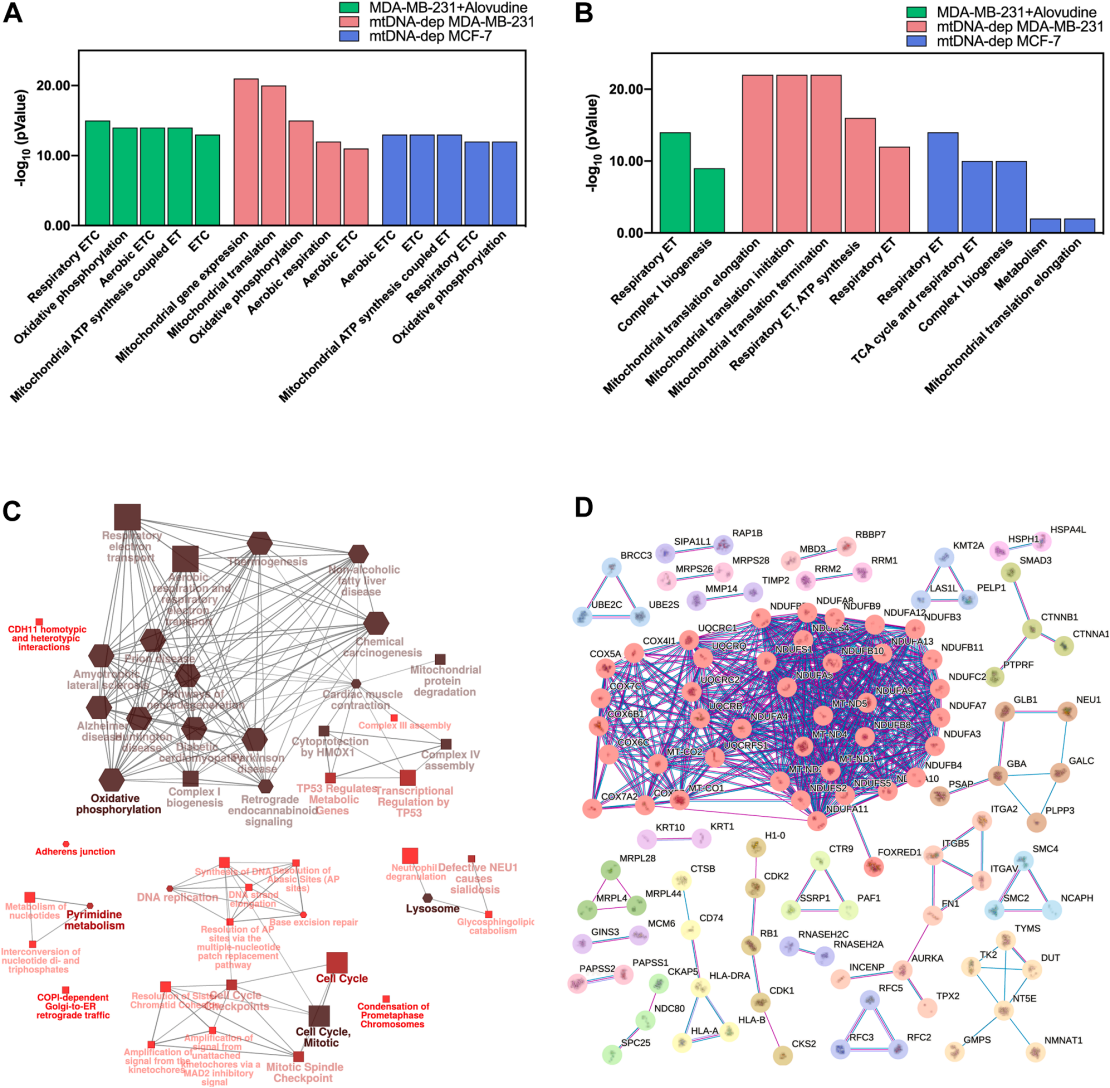
**Bioinformatic analyses of identified proteins.** Functional enrichment analysis of DEPs obtained by String. The five most significantly (*p* < 0.05) enriched terms in *(A)* Biological processes and *(B)* Reactome pathways are presented. The complete list of enriched terms is reported in Supplemental Table S2. *C,* visualization of Reactome (*hexagons*) and KEGG (*rectangles*) pathways characterizing the DEPs of in Alovudine-treated cells obtained by Cytoscape. The node size is proportional to the number of proteins, and the node color depicts the enrichment significance (ranging from *red* = *p*-value < 0.05, to *dark red* = *p*-value < 0.005, and *dark brown* = *p*-value < 0.0005). The most significant pathway of a group is considered to be the leading term and it is highlighted in the network. *D,* protein-protein interaction analysis of DEPs in Alovudine-treated cells obtained by String. The *circles* represent the identified proteins, and the edges represent protein–protein interactions (*blue lines* = interactions from curated databases, *pink lines* = experimentally determined interactions, *purple lines* = protein homologs found interacting in other organisms). DEP, differentially expressed protein.

GO analysis revealed that the majority of DEPs across all 3 cell models were enriched for mitochondrial processes, with electron transport chain (ETC), OXPHOS, mitochondrial ATP synthesis coupled to electron transport (ET), mitochondrial translation and mitochondrial gene expression among the most significantly represented (Fig. 3*A* and Supplemental Table S2). Pathway enrichment highlighted respiratory ET as a top feature of Alovudine-treated cells, whereas mtDNA-depleted MDA-MB-231 cells were particularly characterized for mitochondrial translational processes and mtDNA-depleted MCF-7 cells for respiratory ET (Fig. 3*B*; Supplemental Figs. S3–S5). Moreover, Cytoscape pathway analysis linked DEPs in Alovudine-treated cells mainly to OXPHOS, in addition to lysosomal function and cell cycle pathways (Fig. 3*C*). PPI mapping using STRING revealed a large ETC centered subnetwork (n = 41) among DEPs from Alovudine-treated cells, in addition to a smaller adhesion/migration subnetwork (n = 7) containing integrins and fibronectin (Fig. 3*D*). Furthermore, both mtDNA-depleted cell PPI networks displayed two major clusters: one enriched for mitochondrial translation components and the other for ETC subunits, highlighting coordinated disturbances in mitochondrial gene expression and respiratory function following mtDNA depletion (Supplemental Fig. S5).

### Distinct Non-mitochondrial Responses Across mtDNA Depletion Models

To contextualize our findings with respect to the non-mitochondrial proteome, we repeated the enrichment analysis after excluding mitochondrial proteins from the dataset using the experimentally identified proteome of the corresponding dataset as the statistical background (Supplemental Table S2). The enrichment was predominantly observed for GO Cellular Component terms. In Alovudine-treated cells, the most significantly enriched cellular components included the extracellular matrix, collagen-containing extracellular matrix, lysosomal lumen, and the intrinsic component of the plasma membrane. In addition, KEGG pathway analysis identified significant enrichment of the lysosome pathway. In mtDNA-depleted MDA-MB-231 cells; enrichment was restricted to extracellular matrix- and collagen-containing extracellular matrix-related cellular components. Finally, mtDNA-depleted MCF-7 cells displayed enrichment almost exclusively for plasma membrane-associated cellular components, including plasma membrane, intrinsic component of plasma membrane, cell periphery, and integral component of the plasma membrane.

### Comparative Proteomic Profiling of Alovudine- and EtBr-Induced mtDNA Depletion

Moreover, to see if there are differences related to how mitochondria depletion is accomplished, we compared the proteomic profiles of Alovudine-treated and EtBr-treated MDA-MB-231 cells (Supplemental Table S3). This comparison identified 155 DEPs, of which 83 were upregulated and 72 downregulated in Alovudine-treated cells relative to EtBr-treated cells. No significant functional enrichment was detected when all DEPs were analyzed together, nor among the proteins upregulated in Alovudine-treated cells. In contrast, the downregulated proteins showed significant enrichment for the GO Cellular Component term “integral component of membrane” (Supplemental Table S3). The proteins contributing to this enrichment included several membrane receptors, transporters, and adhesion-associated proteins, such as EPHA2, PTPRF, CDCP1, TFRC, LDLR, SLC38A2, and SLC20A1, indicating preferential modulation of membrane-associated proteins by Alovudine relative to EtBr.

### Genomic Amplification of mtDNA-dependent Proteins in Metastatic Breast Cancer

Remarkably, the majority of proteins downregulated following inhibition of mtDNA synthesis and in mtDNA-depleted cells are encoded by genes that exhibit genomic amplification in metastatic breast cancer patients. Amplification frequencies were extracted separately from the MBC Project, INSERM, TCGA and CPTAC datasets (Supplemental Table S4).

The MBC Project showed markedly higher amplification frequencies for several genes; however, this cohort includes 379 samples from 301 patients and contains both primary and/or metastatic specimens from patients who developed metastatic breast cancer. Therefore, these data were interpreted as derived from a heterogeneous cohort of patients with metastatic breast cancer rather than from a purely metastatic-sample dataset. To determine whether amplification enrichment was supported independently of the MBC Project, we examined the INSERM metastatic dataset, derived from metastatic tumor biopsies collected in the prospective SAFIR01, SAFIR02, SHIVA, and MOSCATO clinical trials, separately. Although amplification rates were generally lower in INSERM, several genes, including NDUFA13, NDUFA4, NDUFA10, NDUFA5, UQCRC1, COX7A2, CD63, COX7C, FTH1, IFITM3, NDUFA3, and SLC38A2, also showed higher amplification frequencies than TCGA primary tumors, providing independent support from a second metastatic breast cancer cohort (Table 3). Interestingly, three additional genes (NDUFA9, NDUFS4, and TFAM), which did not show elevated amplification frequencies in the MBC Project, were nevertheless more frequently amplified in the INSERM cohort than in TCGA primary tumors, further supporting the value of analyzing independent metastatic cohorts separately. By contrast, other genes showed high amplification primarily in the Metastatic Breast Cancer Project dataset and were therefore interpreted more cautiously.

**Table 3:** Amplification Frequencies of Genes Encoding Proteins Commonly Downregulated Following Alovudine Treatment and/or mtDNA Depletion Across Independent Primary (TCGA, CPTAC) and Metastatic (Metastatic Breast Cancer Project, INSERM) Breast Cancer Cohorts

Gene	TCGA Primary (%) (n = 1084)	CPTAC Primary (%) (n = 122)	MBC Project Metastatic (%) (n = 379)	INSERM Metastatic (%) (n = 216)	Independent Support in INSERM^b^
*GBA1*	8.6%	25.4%	52.2%	10.2%	-
*NDUFS2*	9.0%	32.0%	46.5%	8.3%	-
*NDUFB10*	4.2%	10.7%	37.9%	6.5%	-
*ADIRF*	0.6%	0.8%	37.5%	1.4%	-
*NDUFB9*	12.3%	33.6%	27.9%	12.0%	-
*IFITM3*	0.2%	5.7%	27.2%	3.2%	✓
*NDUFA13*	0.7%	2.5%	26.9%	2.3%	✓
*NDUFA3*	1.5%	3.3%	24.6%	4.2%	-
*ATP1B1*	6.9%	32.8%	24.6%	9.7%	✓
*UQCRC1*	—	1.6%	24.3%	0.9%	✓
*FTH1*	0.8%	3.3%	23.3%	3.2%	✓
*UQCRFS1*	2.4%	3.3%	22.9%	2.3%	-
*COX6C*	10.2%	24.6%	20.3%	12.5%	-
*COX6B1*	1.8%	4.9%	19.9%	3.2%	-
*DNASE2*	1.3%	3.3%	19.9%	3.2%	-
*NDUFC2*	6.7%	8.2%	19.6%	10.6%	-
*NDUFB7*	1.1%	4.1%	18.9%	1.4%	-
*MRPS28*	7.8%	14.8%	18.3%	11.6%	-
*CD63*	—	0.8%	17.9%	1.9%	-
*NDUFA11*	0.8%	4.9%	17.9%	2.3%	✓
*NDUFA7*	0.7%	0.8%	16.9%	1.9%	-
*MRPL4*	1.3%	2.5%	16.6%	2.8%	-
*SPAG1*	10.7%	23.8%	15.3%	10.6%	-
*UQCRB*	9.0%	20.5%	14.0%	9.3%	-
*UQCRQ*	0.3%	0.8%	13.0%	0.5%	-
*COX5B*	0.5%	8.2%	11.6%	0.5%	-
*COX7C*	—	0.8%	10.6%	3.2%	✓
*NDUFA8*	0.6%	0.8%	10.6%	0.5%	-
*NDUFA10*	—	—	8.3%	1.4%	✓
*UQCRC2*	3.2%	3.3%	7.3%	0.9%	-
*NDUFA4*	0.6%	1.6%	5.6%	6.0%	✓
*COX7A2*	0.4%	0.8%	5.3%	1.9%	✓
*SLC38A2*	0.6%	1.6%	4.3%	2.8%	✓
*NDUFA12*	0.4%	0.8%	4.0%	0.5%	-
*NDUFA9*	1.7%	5.7%	3.7%	4.2%	✓
*MAK16*	1.8%	6.6%	3.7%	2.3%	-
*TFAM*	1.0%	0.8%	3.0%	3.2%	✓
*NDUFA5*	0.4%	2.5%	2.7%	6.0%	✓
*NDUFS1*	0.3%	1.6%	2.3%	—	-
*NDUFS5*	0.8%	3.3%	2.3%	0.9%	-
*MRPL44*	0.2%	0.8%	1.7%	0.9%	-
*NDUFB3*	0.3%	1.6%	1.3%	0.9%	-
*NDUFS4*	0.2%	0.8%	0.3%	1.4%	✓
*LAMP2*	0.4%	—	—	—	N/A
*RBM3*	0.8%	—	—	—	N/A

The Metastatic Breast Cancer Project includes 379 samples from 301 patients, comprising both primary and metastatic tumor specimens (11).

b Indicates genes with higher amplification frequencies in the INSERM metastatic cohort than in TCGA primary breast cancers.

## Discussion

Understanding and preventing metastatic progression remains a central challenge in modern oncology. In this study, we identify the molecular basis and downstream protein effectors that connect mtDNA integrity to metastatic processes. Metastasis depends on functional mtDNA, intact mitochondrial ribosome activity, and sufficient mitochondrially derived ATP (14, 15, 16, 17). These findings align with our current observation that Alovudine treatment, which inhibits mtDNA synthesis and reduces metastasis (6), is associated with downregulation of gene-regulatory proteins and mitochondrial ribosomal subunits, together with modulation of multiple OXPHOS and TCA-cycle proteins. In addition, we also detected distinct non-mitochondrial responses to mtDNA depletion. The enrichment patterns observed in the non-mitochondrial proteome across the three experimental conditions reveal cell-type-specific responses that extend beyond mitochondrial homeostasis. These findings complement and expand previous observations (18) showing that mitochondrial dysfunction engages broader cellular programs in a tumor and context-dependent manner. Using experiment-specific statistical backgrounds, the non-mitochondrial signatures emerging from the enrichment analysis were predominantly associated with GO Cellular Component terms rather than biological processes. In Alovudine-treated MDA-MB-231 cells, the enrichment of extracellular-matrix and lysosomal components suggests adjustments involving extracellular and membrane-associated compartments. mtDNA-depleted MDA-MB-231 cells showed a more contained pattern, limited to extracellular-matrix–related components, whereas mtDNA-depleted MCF-7 cells were characterized by enrichment of plasma-membrane and cell-periphery components. Overall, these findings indicate that mitochondrial dysfunction is accompanied by non-mitochondrial structural changes whose nature varies according to cellular context. The distinct proteomic signatures observed between Alovudine- and EtBr-induced mtDNA depletion highlight that the mode of mitochondrial impairment shapes, at least in part, the cellular adaptive response. Notably, the findings obtained suggest that, despite producing a comparable mtDNA-deficient phenotype, Alovudine and EtBr differentially affect membrane-associated proteins.

Interestingly, our proteomic analysis uncovered proteins modulated by Alovudine that are linked to metastatic progression, including a subset consistently downregulated in both Alovudine-treated and mtDNA-depleted cells. Importantly, interrogation of metastatic breast cancer datasets shows that many of the corresponding genes of these downregulated proteins are subject to genomic amplifications in cohorts of patients with metastatic disease, supporting their potential role as functionally relevant effectors that connect mtDNA integrity to metastasis. The highest amplification frequencies were generally observed in the MBC Project which, however, includes both primary and metastatic samples. Moreover, differences in cohort composition, including molecular subtype distribution, sample composition, and copy-number calling procedures, as well as the different amplification frequencies reported in the archived and provisional releases of the MBC Project dataset, warrant a cautious interpretation of quantitative differences across datasets and preclude definitive conclusions regarding metastatic specificity. Nevertheless, a subset of candidate genes also displayed higher amplification frequencies in the independent INSERM metastatic cohort than in TCGA primary breast cancers, indicating that these alterations are not unique to the MBC Project. Among the independently supported genes were several components of the mitochondrial respiratory chain, including Complex I subunits (NDUFA3, NDUFA5, NDUFA9, NDUFA10, NDUFA13 and NDUFS4), together with Complex III (UQCRC1) and Complex IV (NDUFA4, COX7A2 and COX7C) proteins. Their recurrent amplification in independent metastatic breast cancer cohorts is consistent with an increased requirement for enhanced ET chain efficiency, increased NAD^+^ regeneration, and sustained ATP production, all of which are recognized contributors to metastatic traits such as cytoskeletal remodeling, migration, and resistance to anoikis (19, 20). Their downregulation upon mitochondrial inhibition in our models further supports their functional relevance within the mtDNA-dependent mitochondrial programme identified in this study.

Both Alovudine treatment and mtDNA-depleted cell generation impair mtDNA replication and respiratory chain assembly, leading to coordinated suppression of OXPHOS proteins. This pattern mirrors previous findings in AML cells treated with Alovudine and in mouse models with defective mtDNA maintenance, where the same respiratory chain components were downregulated (21, 22). Together, these independent datasets reinforce the conclusion that disruption of mtDNA integrity, converges on a conserved suppression of OXPHOS machinery. The overlap between experimentally downregulated proteins and genes amplified in metastatic breast tumors here detected suggests that metastatic cells rely on upregulation (23) of mitochondrial biogenesis and respiratory chain integrity to maintain fitness under the metabolic pressures of dissemination. In this context, Alovudine appears to target a metabolic dependency that metastatic cells reinforce through genomic amplification.

Beyond classical OXPHOS components, the amplification of genes such as FTH1, SLC38A2, IFITM3, and CD63 points to a broader adaptive program supporting metastatic competence that encompasses both metabolic reprogramming and membrane-associated remodeling. FTH1, which contributes to iron homeostasis and ferroptosis susceptibility (24), has been implicated in oxidative stress adaptation, breast cancer progression, and metastatic competence (23). Likewise, the amino acid transporter SLC38A2 supports glutamine uptake and mTOR-dependent metabolic reprogramming, processes increasingly recognized as essential for metastatic colonization and survival in foreign microenvironments (25). IFITM3 and CD63, both membrane-associated proteins, contribute to a distinct layer of metastatic adaptation. IFITM3 promotes epithelial-to-mesenchymal transition, invasion, metastasis, and poor clinical outcome, particularly in triple-negative breast cancer (26, 27), whereas CD63 has been implicated in membrane trafficking, extracellular vesicle biology, and metastatic dissemination (28). CD63-dependent signaling promotes tumor–stroma communication (29), therapeutic resistance and invasion, suggesting that its recurrent amplification may contribute to the adaptive phenotype of advanced breast cancer.

Collectively, the frequent amplification of these genes and their coordinated downregulation upon suppression of mtDNA synthesis reveal a convergence between genomic amplification and mitochondrial dependency, implicating mitochondrial bioenergetics, metabolic adaption, and membrane-associated remodeling as actionable vulnerabilities in metastatic breast cancer. Together, our findings demonstrate that Alovudine suppresses a panel of mtDNA-dependent proteins whose encoding genes exhibit recurrent amplification across independent breast cancer cohorts, with independent support from the INSERM metastatic cohort for a subset of candidates. These proteins emerge as promising candidate biomarkers and potential therapeutic targets associated with metastatic breast cancer.

## Data Availability

MS raw data and Proteome Discoverer search files and results have been deposited to ProteomeXchange under the identifier PXD077655.

## Supplemental data

This article contains supplemental data.

## Competing Interest

The authors declare no competing interests.

## Supplemental data

Supplemental Table S1 Proteomics results. For each experimental comparison, two worksheets are provided: “raw,” containing the complete Proteome Discoverer 2.5 output for all identified proteins, and “DEPs,” containing only the differentially expressed proteins used for downstream bioinformatics analyses. A "column description" worksheet provides a detailed explanation of each exported column. The table includes protein IDs, gene names, peptide counts, label-free quantification values, fold-change ratios, *P* values, and FDR-adjusted significance metrics. The “abundance ratio” values are calculated by Proteome Discoverer 2.5 using the replicate-specific abundance ratios from the nested experimental design and are reported as the geometric median of the biological replicate ratios. DEP, differentially expressed protein; FDR, false discovery rate.

Supplemental Table S2 Functional enrichment analysis. Results of the STRING enrichment analysis for all the DEPs performed using experiment-specific statistical backgrounds consisting of all proteins identified in the corresponding LC-MS/MS experiment. The table includes enriched GO terms, STRING clustres, Reactome and KEGG pathways, enrichment scores, FDR–corrected p-values, number of proteins mapped to each term, and associated protein lists. A "Column description" worksheet provides a detailed explanation of each exported column.

Supplemental Table S3 Proteomic data for Alovudine- and EtBr-treated cells and enrichment analysis. "Proteins" worksheet contains the complete Proteome Discoverer 2.5 output for all identified proteins with protein IDs, gene names, peptide counts, LFQ values, fold changes, *P* values, and FDR-adjusted metrics. "Up" and "down" report the differentially expressed proteins (DEPs) upregulated and downregulated, respectively, in Alovudine-treated cells compared with EtBr-treated cells. "Up enrichment" and "down enrichment" worksheets report the STRING functional enrichment analyses performed separately for upregulated and downregulated DEPs using experiment-specific statistical backgrounds. A "column description" worksheet provides a detailed explanation of each exported column.

Supplemental Table S4 Genomic alteration analysis. Dataset-specific amplification frequencies of genes encoding proteins downregulated following Alovudine treatment and/or mtDNA depletion across independent primary and metastatic breast cancer cohorts. The table reports amplification frequencies for each individual dataset (TCGA, CPTAC, Metastatic Breast Cancer Project, and INSERM), together with pairwise two-tailed Fisher's exact test results (odds ratios and *P* values) comparing metastatic and primary cohorts. Dataset-specific frequencies are presented descriptively and interpreted independently rather than as pooled evidence of metastatic specificity.

Supplemental Figures Supplemental Figure S1. **Overview of assays used to characterize mtDNA-depleted MDA-MB-231 cells.** Seahorse XF analysis showing (*A*) oxygen consumption rate (OCR) and (*B*) extracellular acidification rate (ECAR) parameters, (*C*) colony formation assay used to assess clonogenic capacity. Supplemental Figure S2. **Overview of assays used to characterize mtDNA-depleted MCF-7 cells.** Seahorse XF analysis showing (*A*) oxygen consumption rate (OCR) and (*B*) extracellular acidification rate (ECAR) parameters, (*C*) colony formation assay used to assess clonogenic capacity. Supplemental Figure S3. **GO enrichment analysis.** Histograms of the TOP10 enriched GO cellular component and molecular function terms identified using all the DEPs of (*A*) Alovudine-treated MDA-MB-231 cells, (*B*) mtDNA-depleted MDA-MB-231 cells and (*C*) mtDNA-depleted MCF-7 cells. The *y*-axis shows GO terms, and the *x*-axis shows −log10(FDR) enrichment values. GO, Gene Ontology; DEP, differentially expressed protein; FDR, false discovery rate. Supplemental Figure S4. **Enriched pathways analysis.** Visualization of reactome (*hexagons*) and KEGG (*rectangles*) pathways characterizing all the DEPs of (*A*) mtDNA-depleted MDA-MB-231 cells and (*B*) mtDNA-depleted MCF-7 cells by Cytoscape. Node size reflects protein count, and node color indicates enrichment significance (*red to dark brown*, *p* < 0.05 to *p* < 0.0005). The top enriched pathway in each group is highlighted as the leading term. Supplemental Figure S5. **Protein–protein interaction analysis.** Protein–protein interaction networks generated using all the DEPs in (*A*) mtDNA-depleted MDA-MB-231 cells and (*B*) mtDNA-depleted MCF-7 cells obtained by String. Nodes represent proteins, and edges indicate interactions (*blue* = curated databases, *pink* = experimental evidence, *purple* = homolog-based interactions). DEP, differentially expressed protein.
