# Supplementary material for: Alovudine Targets mtDNA-dependent Proteins Encoded by Genes Recurrently Amplified in Metastatic Breast Cancer Cohorts
Source: Mol Cell Proteomics. 2026 Jul 14;25(8):101622. doi: 10.1016/j.mcpro.2026.101622 (PMC13486814; doi:10.1016/j.mcpro.2026.101622)
Supplement: Supplementary file 1 [file mmc5.docx]

**Supplemental Figure S1**

**Supplemental Figure S2**

**Supplemental Figure S3**

**a**

**b**

**c**

**Supplemental Figure S4**

**b**

**a**

**Supplemental Figure S5**

**a**

**b**
